# Draft Genome Sequences of Six Lactobacillus pentosus Strains Isolated from Brines of Traditionally Fermented Spanish-Style Green Table Olives

**DOI:** 10.1128/genomeA.00379-18

**Published:** 2018-05-03

**Authors:** Beatriz Calero-Delgado, Antonio M. Martín-Platero, Antonio J. Pérez-Pulido, Antonio Benítez-Cabello, Carlos S. Casimiro-Soriguer, Manuel Martínez-Bueno, Francisco Noé Arroyo-López, Francisco Rodríguez-Gómez, Joaquín Bautista-Gallego, Antonio Garrido-Fernández, Rufino Jiménez-Díaz

**Affiliations:** a Departamento de Biotecnología de Alimentos, Instituto de la Grasa, CSIC, Campus de la Universidad Pablo de Olavide, Seville, Spain; b Departamento de Microbiología, Universidad de Granada, Granada, Spain; c Centro Andaluz de Biologia del Desarrollo (CABD, UPO-CSIC-JA), Facultad de Ciencias Experimentales (Área de Genética), Universidad Pablo de Olavide, Seville, Spain

## Abstract

Here, we report the genome sequences of six Lactobacillus pentosus strains isolated from traditional noninoculated Spanish-style green table olive brines. The total genome sizes varied between 3.77 and 4.039 Mbp. These genome sequences will assist in revealing the genes responsible for both technological and probiotic properties of these strains.

## GENOME ANNOUNCEMENT

The genus Lactobacillus comprises many species indigenous to food-related habitats, such as dairy products, meats, sausages, and fermented vegetables (1, 2). Moreover, it has been shown that some species have potential probiotic properties, thus providing health benefits to human consumers (3, 4). The Lactobacillus pentosus species is the main inhabitant in the economically important Spanish-style green olive fermentation process (5). This lactic acid bacterium contributes to the preservation of olives by means of the large amounts of lactic acid produced, as well as by producing antibacterial compounds such us bacteriocins (6). Spanish-style green olive fermentation usually relies on a spontaneous, traditional method in which both the olives and the environment are handled in order to favor the development of L. pentosus (7). However, as the use of starter cultures is not a common practice in this fermentation, undesirable microorganisms frequently dominate the environment, and then spoilage of olives may occur. Thus, the genome sequences reported here will be highly valuable not only for designing appropriated starter cultures for olive fermentation but also for deepening the knowledge of potentially probiotic features shown by the L. pentosus species.

Here, the sequenced genomes of six L. pentosus strains (named L. pentosus IG2 to IG7) isolated from the brine of natural Spanish-style green olive fermentation are presented. The genomic DNA from the strains was extracted using a modification of the protein “salting-out” procedure (8). Genome libraries were constructed using a TruSeq DNA PCR-free library preparation kit (Illumina, Inc.), with an insert size of 350 bp, and sequenced at Macrogen, Inc., (Seoul, Republic of Korea) by using an Illumina HiSeq platform with paired-end sequencing of 2 × 101-bp read lengths. The genomes were assembled with Velvet 1.2.10 (9), and parameters were optimized with VelvetOptimiser 2.2.5 (9). The NCBI Prokaryotic Genome Annotation Pipeline (PGAP) was used to annotate the six strains, and the annotation was completed by use of the following protocol: protein-coding genes were predicted using Prodigal v 2.6.3, which is an accurate prokaryotic gene finder (10), and then they were functionally annotated by Sma3s v2 using UniProt bacteria to get a higher sensitivity (11). To annotate noncoding genes, Infernal 1.1.2 (12) was used with Rfam database 13.0 (13). To estimate the number of plasmids appearing in each strain, the contig sequences were compared to all the plasmid sequences from Lactobacillus species available in the RefSeq database.

The total genome sizes ranged from 3.77 to 4.039 Mbp, thus representing the largest sequenced genomes among L. pentosus strains to date, with very similar G+C contents, which varied from 45.78% to 45.98%. We found several plasmids per strain, ranging from 6 in the IG4 strain to 13 in the IG3 strain. The chromosomes encode from 84 to 92 tRNAs and from 5 to 9 rRNAs. These data show the high genome plasticity of this lactic acid bacterial species.

### Accession number(s).

The genome sequences of the six L. pentosus strains have been stored under NCBI BioProject number PRJNA436944, and genome information and the GenBank accession number for each strain are listed in Table 1.

**TABLE 1:** Genome information and GenBank accession numbers

Strain	GenBank accession no.	Size (bp)	No. of contigs	G+C content (%)	No. of protein- coding genes	No. of plasmids	No. of tRNAs	No. of rRNAs
IG2	PVOB00000000	4,033,890	460	45.70	3,946	7	85	8
IG3	PVOA00000000	3,919,445	111	45.80	3,639	13	92	3
IG4	PVNZ00000000	3,806,728	166	45.97	3,522	6	84	5
IG5	PVNY00000000	3,768,924	96	45.98	3,449	10	88	3
IG6	PVNX00000000	3,882,104	187	45.79	3,631	11	88	3
IG7	PVNW00000000	3,802,404	352	45.79	3,675	10	92	8
